# Coinhibitory Molecules in Autoimmune Diseases

**DOI:** 10.1155/2012/269756

**Published:** 2012-09-11

**Authors:** Norihiko Watanabe, Hiroshi Nakajima

**Affiliations:** ^1^Center for Rheumatic Diseases, Saiseikai Narashino Hospital, Narashino 275-8580, Japan; ^2^Department of Allergy and Clinical Immunology, Graduate School of Medicine, Chiba University, 1-8-1 Inohana, Chuo-ku, Chiba, Chiba 260-8670, Japan

## Abstract

Coinhibitory molecules such as CTLA-4, PD-1 and BTLA negatively regulate immune responses. Multiple studies indicate that the deficiency or mutation of coinhibitory molecules leads to the development of autoimmune diseases in mice and humans, indicating that the negative signals from coinhibitory molecules are crucial for the prevention of autoimmunity. In some conditions, the administration of decoy coinhibitory receptors (e.g., CTLA-4 Ig) or mAb against coinhibitory molecules suppresses the responses of self-reactive T cells in autoimmune diseases. Therefore, modulation of coinhibitory signals seems to be an attractive approach to induce tolerance in autoimmune diseases in humans where the disease-inducing self-antigens are not known. Particularly, administration of CTLA-4 Ig has shown great promise in animal models of autoimmune diseases and has been gaining increasing attention in clinical investigation in several autoimmune diseases in humans.

## 1. Introduction

 The immune system has developed multiple mechanisms to prevent harmful activation of immune cells. One such mechanism is the balance between costimulatory and coinhibitory signals delivered to T cells. The B7-1 (CD80)/B7-2 (CD86)-CTLA-4 pathway is the best-characterized inhibitory pathway for T-cell activation [1–3]. Another inhibitory pathway involves programmed death-1 (PD-1), which interacts with PD-L1 (B7-H1) and PD-L2 (B7-DC) and negatively regulates T cell activation [1, 3, 4]. B and T lymphocyte attenuator (BTLA), the third coinhibitory molecule for T-cell activation, is a cell surface molecule with similarities to CTLA-4 and PD-1 [5]. The ligand for BTLA is herpesvirus-entry mediator (HVEM), a TNF receptor family protein, and the ligation of BTLA with HVEM attenuates T-cell activation [6–9]. Since these inhibitory coreceptors inhibit proliferation and cytokine production of T cells *in vitro* and *in vivo*, they are thought to play important roles in maintaining immunological homeostasis and tolerance [10–12]. 

 Autoimmune diseases occur because of a failure of the immune system to maintain nonresponsiveness or tolerance to self-antigens. Accumulating evidence indicates that coinhibitory molecules are key in the prevention of autoimmune diseases, because a defect or a functional mutation in these molecules promotes autoimmunity and polymorphisms of these genes are associated with genetic susceptibility to autoimmune diseases in humans. 

 Once an autoimmune disease developed, whether it is organ specific or nonorgan specific, in most cases corticosteroids and/or immunosuppressants are used for treatment. Refractory autoimmune diseases are sometimes treated with biological agents such as TNF*α* blockers, anti-IL-6 receptor antibody, and anti-CD20 antibody. However, immunosuppressive therapy occasionally causes serious adverse effects such as infection and malignancy. Therefore, novel immunomodulating agents for autoimmune diseases that have fewer adverse effects are desired.

 This review is intended to give an overview of the immunobiology of the coinhibitory molecules and their roles in autoimmune diseases. We also review the advantages and limitations that should be discussed to translate the targeting of coinhibitory pathways into successful therapeutic interventions. 

## 2. CD28/CTLA-4-B7 Pathway in the Regulation of Immune Responses

 Numerous studies have demonstrated the importance of CD28-B7 costimulation for TCR-MHC-mediated T cell activation [13]. The interaction between CD28 on T cells and the B7 family molecules [B7-1 (CD80) and B7-2 (CD86)] on antigen presenting cells (APCs) plays a central role not only in the activation of normal (protective) T cell responses but also for the activation of pathological (self-reactive) T cell responses [1, 14]. CD28 is constitutively expressed on naïve and activated T cells. B7-1 is expressed in low levels on resting APCs and is upregulated with prolonged interaction with T-cells, whereas B7-2 is constitutively expressed and rapidly upregulated on APCs. Thus, B7-2 is likely to be mainly involved in mediating initial T cell activation, while B7-1 may play an important role in propagating the immune responses. After activation, T cells express CTLA-4 (CD152), which has higher affinity for B7-1 and B7-2 than CD28 does [15, 16]. Engagement of CTLA-4 delivers negative signal into T cells, resulting in inhibition and/or termination of T cell responses. CD28-B7 interactions are also important for the expansion and maintenance of CD4^+^CD25^+^ Tregs [17].

## 3. Roles of CTLA-4 Pathway in the Maintenance of Self-Tolerance

 A defect in the negative signals from coinhibitory molecules may lower the threshold of autoreactive lymphocyte activation and thus may lead to the development of autoimmune diseases. This notion has been first evidenced by the autoimmune phenotype or lymphocyte hyperreactivity in mice lacking CTLA-4. CTLA-4-deficient mice rapidly develop a lymphoproliferative disease with multiorgan lymphocytic infiltration and tissue destruction and die by 3-4 weeks of age [18, 19]. In humans, CTLA-4 has been suggested to be associated with various autoimmune diseases including Grave's disease, autoimmune hypothyroidism, type I diabetes, systemic lupus erythematosus (SLE), and celiac disease [20–24]. Interestingly, Cunninghame Graham et al. have shown that although the 3′ flanking region of CTLA4 is an important region for association to both SLE and Graves' disease, the pattern of association to SLE is distinct from that seen in Graves' disease and the variants contributing to the association in SLE are more distal to CTLA4 than those in Graves' disease [23]. These findings suggest that CTLA-4 plays critical roles in the prevention of autoimmunity in multiple organs through multiple mechanisms. 

## 4. Blockade of CD28-B7 Pathway as a Therapy for Autoimmune Diseases

 It is anticipated that therapies directed against the B7 molecules would selectively affect T cells that are in the process of antigen-induced activation but would not affect resting T cells. Thus, in patients with autoimmune diseases, blockade of B7-CD28 interactions might preferentially inhibit lymphocytes that are in the process of responding to self-antigens without affecting resting T cells that recognize other antigens.

 To develop the agents that would block signaling through CD28, investigators have taken advantage of the fact that CTLA-4 binds B7-1 and B7-2 with much higher affinity than CD28 does [15, 16]. A fusion protein consisting of the extracellular domain of CTLA-4 and the constant region of IgG blocks the interaction between B7 molecules and CD28 and thereby inhibits T-cell activation [16]. This fusion protein, designated CTLA-4 Ig, has been used successfully in mice to block T cell responses, to inhibit B-cell differentiation into plasma cells, to facilitate organ transplantation, and to induce anergy to self-antigens [25–27]. As for treatment of autoimmune disease models, CTLA-4 Ig treatment prevents autoantibody production, reduces the severity of lupus nephritis, and prolongs survival in NZB/NZW F1 mice [28]. CTLA-4 Ig treatment also prevented experimental autoimmune encephalomyelitis (EAE) induced by either active immunization or adoptive transfer of activated antigen-specific T cells [29, 30].

*In vivo* studies using anti-CD80 mAbs and anti-CD86 mAbs have suggested that CD80 and CD86 differentially regulate the development of autoimmune disease. Actively induced EAE is ameliorated by treatment with anti-CD80 mAb and is exacerbated by treatment with anti-CD86 mAb [31]. On the other hand, the development of diabetes in NOD mice, a model for insulin-dependent diabetes mellitus (IDDM), is exacerbated by treatment with anti-CD80 mAb and is blocked by treatment with anti-CD86 mAb [32]. In rheumatic disease models, both anti-CD80 mAb and anti-CD-86 mAb are required to suppress disease manifestations in lupus mice [33] or collagen-induced arthritis (CIA) models [34]. These conflicting results can be explained by the influence of the timing of the treatment or the differences of the pathology of disease models. The fact that these treatments block the signaling not only from CD28 but also from CTLA-4 may explain the reason why the treatment with these agents exacerbates autoimmune responses in certain situations. 

## 5. Clinical Application of CTLA-4 Ig for Human Autoimmune Diseases

Based on the encouraging results in murine models, the efficacy of CTLA-4 Ig has been examined in patients with autoimmune diseases. Abatacept is a fusion protein composed of the Fc fragment of a human IgG1 linked to the extracellular domain of CTLA-4 [35]. Abatacept has shown efficacy in a broad spectrum of rheumatoid arthritis (RA) patients from early stage to refractory diseases that are resistant to TNF blockers [36, 37]. Abatacept treatment results in significant improvement in the signs and symptoms of RA including the inhibition of the structural damage [38]. Abatacept has also demonstrated efficacy in patients with juvenile idiopathic arthritis (JIA) who have not responded to traditional DMARDs or TNF blockers [39].

 In addition to RA and JIA, Abatacept has shown clinical efficacy in patients with psoriasis in a phase I trial [40]. Abatacept has also demonstrated efficacy in patients with psoriatic arthritis including those who exhibit an inadequate response to TNF blockers [41]. In a study conducted on SLE patients, the efficacy of Abatacept on musculoskeletal manifestations has also been demonstrated [42]. 

 Belatacept (LEA29Y) is another human CTLA-4 Ig that differs from Abatacept by substitution of two amino acids, which confers a stronger binding avidity to B7 and a greater inhibition of T-cell activation. The treatment with Belatacept has been shown to be as effective as cyclosporine in preventing acute rejection after renal transplant and in helping preserve glomerular filtration rate [43, 44]. Phase I/II clinical trial of multiple-dose of Belatacept versus Abatacept versus placebo in RA has revealed preliminary efficacy of Belatacept in the treatment of RA. 

## 6. PD-1-PD-L1/PD-L2 Pathway Inhibits T Cell Activation 

 PD-1 (CD279) is another coinhibitory receptor belonging to CD28 family [45, 46]. PD-1 is expressed on activated T cells, B cells, regulatory T cells, and monocytes and binds to two ligands of the B7 family, PD-L1 (B7-H1), and PD-L2 (B7-DC) [47, 48]. The ligation of PD-1 with these ligands inhibits proliferation of CD4 T cells and CD8 T cells by arresting the cell cycle [49]. Whereas PD-L2 expression is mostly restricted to innate immune cells such as dendritic cells (DCs) and macrophages, PD-L1 is expressed not only on hematopoietic cells including T cells, B cells, mast cells, DCs, monocytes, and macrophages but also on several parenchymal tissues including the vascular endothelium and epithelium of multiple organs [1, 4, 46, 50, 51]. The expression of PD-L1 in nonhematopoietic cells suggests that PD-L1 suppresses self-reactive T cells or B cells in peripheral tissues and may regulate inflammatory responses in the organs. 

 Unlike CTLA-4-deficient mice, PD-1 deficiency leads to autoimmune disorders later in life. PD-1-deficient mice on a C57BL/6 background spontaneously develop lupus-like glomerulonephritis and proliferative arthritis [52]. In addition, PD-1-deficient mice on a BALB/c background develop dilated cardiomyopathy [53], which is associated with the production of autoantibody against cardiac troponin I, and die of congestive heart failure [54]. 

## 7. Blockade of PD-1-PD-L1/PD-L2 Pathway in Autoimmune Diseases

Targeting PD-1 with an agonist could be an alternative approach for the treatment of autoimmune diseases. However, so far more effort has been directed at blocking this pathway to relieve PD-1-mediated immune suppression in the context of chronic viral infection and tumor immunotherapy [4]. The first study for blockade of PD-1 pathway in autoimmune diseases has reported that blockade of PD-1 and PD-L1, but not of PD-L2, accelerates the onset of the diabetes in NOD mice [55]. On the other hand, blockade of PD-L2 but not of PD-L1 augments EAE in C57BL/6 mice with minimal and delayed expression of PD-L2 in the central nervous system [56]. In contrast, blockade of PD-L1 but not of PD-L2 significantly increases the incidence of EAE in BALB/c mice upon immunization with MOG peptide [57]. These results suggest that PD-L1 and PD-L2 differentially regulate the susceptibility and chronic progression of EAE in a strain specific manner.

 Thus far, more than 30 single nucleotide polymorphisms (SNPs) have been identified within *PD-1* gene. Many reports have highlighted that some regulatory SNPs in *PD-1* might affect the expression and transcription of the gene [58, 59]. The SNPs have been studied as a part of attempts to identify the pathogenesis of several autoimmune diseases including SLE [58–60] and RA [61]. For instance, Prokunina et al. have shown that one intronic SNP in *PD-1* gene, which alters a binding site for the runt-related transcription factor 1 (RUNX1) located in an intronic enhancer, is associated with development of SLE [58]. SNPs in the *PD-1* gene have also been studied in multiple sclerosis (MS) [62], ankylosing spondylitis (AS) [63], and Graves' disease [64]. In Japanese and Filipino populations, a higher frequency of a specific SNP in the *PD-1* gene has been demonstrated in patients with subacute sclerosing panencephalitis (SSPE) [65]. Currently, two anti-PD-1 mAbs are undergoing Phase II trials to determine the efficacy against tumors. One of them, MDX-1106, has shown clinical efficacy in renal cell carcinoma and melanoma without serious toxicity [66]. The efficacy of anti-PD-1 mAb or PD-1 Ig treatment in the patients with autoimmune diseases is needed to be elucidated in the further basic and clinical studies.

## 8. BTLA-HVEM Pathway Is the Third Inhibitory Pathway for Lymphocyte Activation 

 BTLA (CD272) is the third inhibitory coreceptor, which has been identified as an inhibitory receptor on CD4^+^ T cells and B cells with similarities to CTLA-4 and PD-1 [5]. Later analyses have revealed that BTLA is expressed not only on CD4^+^ T and B cells but also on a wide range of hematopoietic cells including CD8^+^ T cells, NKT cells, NK cells, macrophages, and dendritic cells at various levels [9, 67–69]. Moreover, it has recently been demonstrated that BTLA is highly expressed on follicular B helper T cells (Tfh cells) [70]. The ligand for BTLA is the TNF receptor family member HVEM [7, 8, 71], which is broadly expressed on hematopoietic cells, including T cells, macrophages, and DCs [71]. Ligation of BTLA induces its tyrosine phosphorylation and SHP-1/SHP-2 association and then attenuates IL-2 production and proliferation of T cells [5, 72]. These findings suggest that BTLA functions as an inhibitory coreceptor through the interaction with HVEM. 

## 9. Relevance of BTLA-HVEM Pathway in Autoimmune Diseases

 Recent analyses suggest that BTLA is crucial for dampening immune responses. BTLA-deficient mice exhibit enhanced specific antibody responses and sensitivity to EAE [5], rapid rejection of partially MHC-mismatched cardiac allograft [73], and acceleration of experimental colitis [74]. We have shown that the deficiency of BTLA also causes the breakdown of self-tolerance, resulting in the development of an autoimmune hepatitis- (AIH-) like disease and lymphocytic infiltration in multiple organs [75]. We have also shown that BTLA plays a protective role in autoimmune diseases in MRL-lpr mice and that AIH-like disease develops in BTLA-deficient mice even in the absence of Fas-dependent signaling [76]. 

 It has been reported that combined treatment with anti-BTLA mAb and CTLA-4 Ig in a fully MHC-mismatched islet transplant model induces donor-specific tolerance [77]. The effect of combined treatment with anti-BTLA mAb and anti-PD-1 mAb on autoimmunity has also been examined in NOD mice [78]. This study has shown that the onset of diabetes is delayed when anti-BTLA mAb is given to 10-week-old NOD mice. In addition, anti-BTLA mAb inhibits anti-PD-1 mAb-induced acceleration of diabetes in NOD mice [78]. Moreover, Ishida et al. have demonstrated that anti-BTLA mAb treatment during the induction phase of ragweed-induced experimental conjunctivitis significantly increases eosinophil infiltration and Th2 cytokine production from T cells [79]. These data support the notion that BTLA pathway is involved in the regulation of immune responses not only to self-antigens but also to non-self-antigens and suggest the efficacy of the modulation of BTLA pathway in multiple immune diseases.

 The role of HVEM-BTLA pathway in the pathogenesis of autoimmune diseases in humans is still largely unknown. However, Lin et al. have recently shown the significant association between the SNP (C+800T) in the BTLA gene with the RA susceptibility in a Chinese population [80]. We have also shown that a functional polymorphism of BTLA gene, which lacks the inhibitory activity, is significantly associated with RA susceptibility in a Japanese population [81]. As of today, no active clinical trial of agents targeting BTLA-HVEM pathway is reported. We assume that the enhancement of BTLA signaling is applicable to the treatment of autoimmune diseases and that the blockade of this pathway may be useful for the treatment of the reduced immune responses against tumors or infection. 

## 10. Concluding Remarks

 The manipulation of signals exchanged between APCs and T-cells has considerable clinical relevance. T cell responses are context-dependent and are influenced by signals from their environment through a variety of receptor-ligand interactions. These signals amplify and modify the original TCR signal received by antigenic stimulation in T cells, regulate expansion and differentiation of activated T cells, or control effector functions in a particular environment. The agents regulating the signals through coinhibitory molecules including CTLA-4, PD-1, and BTLA have the potential to regulate autoimmunity and responses to tumors and chronic infections. Coinhibition, by its very nature, is not antigen specific, and therefore will not be specific for self-reactive T cells. However, because immune responses in the various autoimmune diseases and in normal responses may differ in their requirement for costimulatory and coinhibitory signals, selective costimulation blockade, or coinhibition boost by the administration of the agents against coreceptors may have a therapeutic potential in the treatment of autoimmune diseases. 
